# Effectiveness of complete decongestive therapy for upper extremity breast cancer-related lymphedema: a review of systematic reviews

**DOI:** 10.1007/s12032-024-02421-6

**Published:** 2024-10-23

**Authors:** Laura Gilchrist, Kim Levenhagen, Claire C. Davies, Linda Koehler

**Affiliations:** 1 Doctor of Physical Therapy Program, St. Catherine University, St. Paul, MN USA; 2 Department of Physical Therapy and Athletic Training, Saint Louis University, Saint Louis, MO USA; 3 Nursing and Allied Health Research Office, Baptist Health Lexington, Lexington, KY USA; 4 Department of Family Medicine and Community Health, Division of Physical Therapy & Rehabilitation Medicine, University of Minnesota, Minneapolis, MN USA

**Keywords:** Oncology, Rehabilitation, Intervention, Evidence-based practice

## Abstract

Breast cancer-related lymphedema (BCRL) remains a challenging condition impacting function and quality of life. Complete decongestive therapy (CDT) is the current standard of care, necessitating a comprehensive review of its impact. This paper presents a systematic review (SR) of SRs on CDT’s efficacy in BCRL, and the components of manual lymph drainage (MLD) and exercise. A literature search yielded 13 SRs published between January 2018 and March 2023 meeting inclusion criteria, with varied quality ratings based on the AMSTAR II. A sub-analysis of CDT investigated the within group effect size estimations on volume in different stages of lymphedema. While a moderate quality SR indicated support for CDT in volume reduction, other SRs on the topic were of critically low quality. Larger effect sizes for CDT were found for later stage BCRL. The impact of MLD as a component of CDT demonstrated no additional volume benefit in a mix of moderate to low quality SRs. Similarly, exercise’s role in volume reduction in CDT was limited, although it demonstrated some benefit in pain and quality of life. A rapid review of trials published January 2021–March 2023 reinforced these findings. Variability in CDT delivery and outcomes remained. These findings underscore the need to standardize staging criteria and outcome measures in research and practice. Future research should focus on refining interventions, determining clinically important differences in outcomes, and standardizing measures to improve evidence-based BCRL management. Current evidence supports CDT’s efficacy in BCRL. MLD and exercise as components of CDT have limited support for volume reduction.

**Supplementary Information:**

The online version contains supplementary material available at 10.1007/s12032-024-02421-6.

## Introduction

Upper extremity lymphedema related to cancer can be described as a condition where heaviness, numbness, and swelling occur due to the accumulation of abnormal tissue fluid beneath the skin, triggered by cancer or its treatments. Breast cancer-related lymphedema (BCRL) is the most common type affecting the upper extremity and trunk, with a prevalence of 42% at 18 months post-surgery, and an overall incidence of 21.9% (95% CI, 19.8–24.0%) [1]. While this is a common condition among breast cancer survivors, there are variations in the measurement of and treatment for this condition. Currently, multiple staging classifications for lymphedema exist. The International Society of Lymphology (ISL) staging system is one commonly used set of definitions that identifies tissue quality and volume changes to determine stage [2]. In addition, many researchers have described their populations with more subjective terms, such as mild, moderate, and severe lymphedema with a wide variety of definitions. These differences in classification systems can hinder efforts to understand the efficacy of interventions.

Effective interventions for BCRL are needed as function and quality of life (QOL) can suffer in survivors of breast cancer with lymphedema [3]. Internationally recognized as the standard intervention for lymphedema of different types, complete decongestive therapy (CDT) encompasses manual lymphatic drainage (MLD), compression, exercise, skin care, and education [4]. CDT consists of two phases: an intensive phase, aimed at reducing limb volume in diagnosed lymphedema cases, and a maintenance phase. The intensity and duration of CDT can vary depending on the severity or stage of lymphedema. The majority of CDT evidence is in BCRL with many systematic reviews (SRs) in the past few years focusing on the impact of this treatment. Given the diversity in measurement methods, lymphedema stages, and treatment components across studies, making sense of the evidence presents a challenge.

One consideration for understanding the impact of an intervention, that has not always received sufficient attention, is the amount of change required to be meaningful. In 2018, Tidhar et al. [5] aptly noted that “There is inconsistency between studies as to what is considered a change in volume and, moreover, what is meaningful change.” While statistical significance in a clinical trial can detect differences in outcomes, it is also important to consider if that difference is clinically important or meaningful. Change may be measured after an intervention, but the error inherent in a measure needs to be considered when determining if that change is real or may be a result of measurement error. Thus, the minimally detectable change (MDC) is related to the standard error associated with a measure that has been taken repeatedly. The MDC for volume in the upper extremity was estimated to be 150 ml when using circumferential measures by Taylor et al. [6], indicating that a change of more than 150 ml would be needed to ensure a change was due to the intervention. This study was undertaken with 2 raters, allowing for differences between clinicians in measuring outcomes. Tanori-Tapia [7] in 2020 indicated that a 2.39% (42.9 mL) volume change of the arm would be needed to be confident that the difference was beyond measurement error when only a single rater was used.

In addition to understanding if a change is beyond measurement error, the amount of difference needed for an outcome to be meaningful to people with the condition is essential to understand. In 2020, Devrieze et al. [8] investigated the association between excess arm volume, fibrosis, and a lymphedema specific QOL scale. In 185 patients with BCRL (stages I, IIa, IIb) they found no significant association between excess arm volume and the Lymphedema Functioning Disability and Health Questionnaire for Upper Limb domains or total score. This calls into question if a change in volume is most meaningful to patients or if some additional characteristic is more impactful. Thus, the minimally clinically important difference for volume has yet to be established to our knowledge.

The literature is rife with uncertainties about the best interventions for lymphedema to bring real and noticeable change to patients. As CDT is the standard of care for this condition, it is critical to understand its impact. Thus, a comprehensive understanding of the evidence on CDT was requested by the American Cancer Society/Lymphology Association of North America (LANA) 25th Summit planning committee to inform clinical practice and future research directions. As the majority of the evidence was completed in BCRL, this author group focused the review on this population. Hence, the objectives of this review project were to evaluate the effectiveness of (1) CDT in BCRL and (2) the specific roles of MLD and exercise within CDT for this population. As volume is often the most direct measure of lymphedema taken in rehabilitation clinics and the majority of the literature uses this as an outcome, we focused our initial review on volume with consideration of other outcomes as relevant. Compression, another integral component of CDT, as well as other modalities that could be used in the treatment of lymphedema were evaluated separately by a different presentation group, and thus not included in this paper. As a large number of SRs have been published in recent years covering interventions for BCRL, a review of SRs was conducted. This was followed by a rapid review of literature published more recently than the SRs to determine if this new literature would meaningfully impact their conclusions. We also sought to outline recommendations for future research based on noted gaps in the literature.

## Methods

A literature search for SRs related to the impact of CDT in BCRL published between January 2018 and March 2023 was undertaken. The SRs were extracted from a larger literature search on interventions for upper quadrant lymphedema. The following search terms were used: “Lymphedema* OR lymphoedema* OR elephantiasis.” We excluded “Filariasis OR filarial OR parasite* OR Congenital OR hereditary”. The following databases were searched: PubMed, CINAHL, EMBASE, Cochrane, PEDRO, Scopus, and SportDiscus. In the initial title and abstract screening process for the larger project, articles on upper extremity cancer-related lymphedema were tagged if they investigated rehabilitation interventions and also if they were SRs. For this project, this list of articles was reviewed independently by two authors for inclusion or exclusion based on the following criteria:

- Inclusion: non-surgical or non-pharmacologic interventions for upper quadrant lymphedema, cancer-related lymphedema, human subjects, adults.
- Exclusion: lower extremity or pelvic lymphedema, non-cancer related, animal studies, case reports, narrative reviews, perspectives or opinion papers, conference abstracts, compression studies.

From this literature pool, SRs of BCRL investigating the impact of CDT or one of its components were identified for further examination. During the course of the project, it was identified that another group at the Summit would be reviewing the literature related to the use of compression in CDT, so these SRs were identified and shared with that particular group. These SRs were then excluded from further consideration.

Each included SR was reviewed for quality by the entire author group using the AMSTAR II criteria [9]. The AMSTAR II has 7 criteria identified as critical, but the creators indicate that these can be changed to fit the purpose of a particular review [9]. We retained 6 of the 7 criteria as critical, moving the item on justification for exclusion of individual studies (question 7) from a critical to non-critical item. Authors came to consensus on the rating for each AMSTAR II item and then used the critical and non-critical items to rate the overall quality of the SRs. We defined a partial yes as a non-critical flaw for item 4 on comprehensive search strategy. To meet a “high” quality metric, a review must have no critical weaknesses and one or fewer non-critical weakness. For “moderate” a review must have no critical flaws but may have more than one non-critical weakness. “Low” quality reviews had one critical flaw with or without non-critical flaws. And “critically low” reviews had more than one critical flaw with or without non-critical flaws.

The quality assessments were investigated using descriptive analyses. The summary of the effectiveness of CDT and the specific roles of MLD and exercise within CDT for BCRL are described for volume and other identified outcome measures. A sub-analysis investigated the within group effect size estimations of CDT SR’s that included different stages of lymphedema. Based on the SRs, the evidence for CDT in total and MLD and exercise as components of CDT was reviewed with consideration of SR quality. Recommendations on the interventions were then made with consensus of the author group.

Due to the time required to write and publish SRs, there is the potential for new literature to have been published that could influence the findings. Therefore, a rapid review of additional literature was also undertaken on the same topics using the same search criteria, with the exception of including of clinical studies instead of SRs. This rapid review encompassed literature published between January 1, 2021 and March 31, 2023, as the literature in the included SRs only represented articles published through 2020. The quality of the new literature was not rated, and literature was only reviewed to determine if these additional studies agreed or disagreed with the major findings of the included SRs or instead may indicate a potential to change the recommendations. The articles identified in this literature update were reviewed by all group members and consensus as to their potential impact on the literature base was determined by all authors.

## Results

### State of the systematic reviews on BCRL

**Fig. 1:**
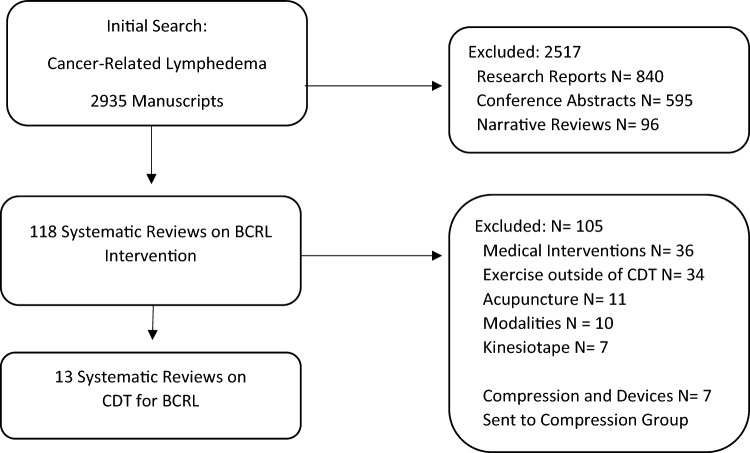
.

During the review period of 2018–March 2023, 118 SRs on non-surgical or non-pharmacologic intervention for BCRL were identified (Fig. 1). The most common reason for exclusion was that the intervention review did not cover CDT as an intervention. This included 34 reviews of exercise outside of CDT, 11 reviews of acupuncture, 7 reviews on kinesiotaping, and 10 reviews of modalities including laser, photobiomodulation, and extracorporal shock wave treatment. In addition, 7 reviews on compression were shared with the Summit group discussing this component of CDT and excluded from further consideration. The search yielded a total of 13 SRs on the topic of CDT in total or MLD or exercise as components of CDT.

Of the 13 SRs included in this literature summary, only 3 received a rating of moderate quality using modified AMSTAR II criteria [4, 10, 11] (Table 1). Three SRs were of low quality [12–14] and 7 were of critically low quality [15–21]. Common critical issues included: a lack of the use of risk of bias information in the discussion of findings (Q13); problematic meta-analysis approaches (Q11); and a lack of comprehensiveness in the literature search (Q4). For example, 2 of the SRs did not complete a risk of bias assessment with a standardized measure (Q9). In addition, most SRs had non-critical flaws in not reporting the sources of funding for included studies (*n* = 12) or lacking multiple, independent bias raters (*n* = 6) and data extractors (*n* = 9). Twelve of the 13 reviews investigated the impact of interventions on limb volume, 5 included the impact on symptoms, and 6 on influence of intervention on QOL. The 13 SRs included 56 unique studies, with individual SRs including a range of 6 to 35 reports. No single study was included in all of the SRs. (Supplemental Table).

**Table 1:** Results of the AMSTAR-2 assessments

Author	Q1	Q2	Q3	Q4	Q5	Q6	Q7	Q8	Q9	Q10	Q11	Q12	Q13	Q14	Q15	Q16	Rating
Jeffs [10]	Y	Y	Y	Y	N	N	Y	Y	Y	N	NA	NA	Y	Y	NA	Y	M
Lin [4]	Y	Y	Y	PY	Y	Y	N	PY	Y	N	Y	Y	Y	Y	Y	Y	M
Muller [11]	Y	Y	Y	PY	Y	N	N	N	Y	N	NA	NA	Y	Y	NA	Y	M
Qiao [12]	Y	PY	Y	PY	N	Y	N	N	Y	N	Y	Y	Y	N	Y	Y	L
Thompson [13]	PY	PY	Y	PY	Y	N	Y	PY	Y	N	NA	NA	Y	Y	NA	Y	L
Wanachi [14]	Y	PY	Y	PY	N	N	PY	PY	Y	N	NA	NA	Y	N	NA	Y	L
Baumann [15]	PY	PY	Y	N	Y	N	N	PY	Y	N	NA	NA	N	N	NA	Y	CL
Liang [16]	Y	PY	Y	PY	Y	Y	N	PY	PY	N	Y	N	N	Y	Y	Y	CL
Lin [17]	Y	PY	Y	PY	Y	Y	N	N	N	N	N	Y	Y	N	N	Y	CL
Lytvyn [18]	PY	Y	Y	PY	Y	U	Y	PY	Y	Y	N	Y	Y	Y	N	Y	CL
Naik [19]	PY	PY	Y	PY	N	N	N	PY	Y	N	NA	NA	N	N	NA	Y	CL
Rangon [20]	Y	Y	Y	PY	Y	Y	N	PY	Y	N	N	Y	N	Y	Y	Y	CL
Smile [21]	N	N	N	N	N	N	N	N	N	N	NA	NA	N	N	NA	Y	CL

*AMSTAR-2* Assessing the Methodological Quality of Systematic Reviews 2, *Y *Yes, *N *No, *PY* Partial Yes, *NA* Not applicable, *U* Unclear, *CL* Critically low, *L* Low, *M* Moderate, *H* High

Grey columns indicate the critical domains for the AMSTAR-2 reviews

Q1: Did the research questions and inclusion criteria for the review include the components of PICO?

Q2: Did the report of the review contain an explicit statement that the review methods were established prior to the conduct of the review and did the report justify any significant deviations from the protocol?

Q3: Did the review authors explain their selection of the study designs for inclusion in the review?

Q4: Did the review authors use a comprehensive literature search strategy?

Q5: Did the review authors perform study selection in duplicate?

Q6: Did the review authors perform data extraction in duplicate?

Q7: Did the review authors provide a list of excluded studies and justify the exclusions?

Q8: Did the review authors describe the included studies in adequate detail?

Q9: Did the review authors use a satisfactory technique for assessing the risk of bias (RoB) in individual studies that were included in the review?

Q10: Did the review authors report on the sources of funding for the studies included in the review?

Q11: If meta-analysis was performed did the review authors use appropriate methods for statistical combination of results?

Q12: If meta-analysis was performed, did the review authors assess the potential impact of RoB in individual studies on the results of the meta-analysis or other evidence synthesis?

Q13: Did the review authors account for RoB in individual studies when interpreting/discussing the results of the review?

Q14: Did the review authors provide a satisfactory explanation for, and discussion of, any heterogeneity observed in the results of the review?

Q15: If they performed quantitative synthesis did the review authors carry out an adequate investigation of publication bias (small study bias) and discuss its likely impact on the results of the review?

Q16: Did the review authors report any potential sources of conflict of interest, including any funding they received for conducting the review?

### Complete decongestive therapy (CDT)

Five SRs reviewed the impact of CDT on arm volume as a primary outcome [10, 18–21]. Only one SR was of moderate quality [10], while the remaining SRs were of critically low quality [18–21]. (Table 2) There was wide variability in the number of studies included in each SR ranging from 8 to 23. When the inclusion of articles (*n* = 56) across SRs was analyzed, (Supplemental Table) no studies were included in all of the SRs and only 3 studies were included in 4 out of 5 SRs. Of the 56 articles, 42 were included in only 1 SRs on CDT. All of the SRs included studies with mild, moderate, and severe lymphedema; with only one [21] considering severity as a sub-analysis. Three of the 5 SRs found evidence to support CDT for volume reduction in BCRL, including the one SR of moderate quality [10]. This SR indicated that there was “weak” evidence to support decongestive treatment for volume reduction, based on inclusion of 8 studies. They concluded that additional randomized, controlled trials were needed [10].

**Table 2:** Systematic reviews on effect of complete decongestive therapy on volume outcomes

Author AMSTAR II rating	Time limit of retrieval	Trials number (sample size)	Experimental intervention	Control intervention	Outcomes investigated (number of studies)	Meta-analysis completed	Main conclusion	Quality assessment tool
Jeffs [10] moderate	Inception to July 2016	5 (196)	Decongestive lymphedema treatment	Another form of lymphedema treatment, placebo, or no treatment	Volume (5); QOL (3); symptoms (1); arm function (2); perceived benefit (2)	No	Weak evidence for the effect of CDT to reduce arm volume	JBI SUMARI
Lytvyn [18] critically low	Inception through October 2019	36 (1651)	Conservative care including CDT	Standard Treatment	Volume (5); Symptoms (5); Function (6); Pain (8); QOL (5)	Yes	Compared to standard care, there is little to no difference in lymphedema volume from CDT (SMD 0.07; 95% CI − 0.29, 0.43)	Cochrane risk of bias assessment tool
Niak [19] critically low	January 2004–March 2020	14 (878)	Various Physiotherapy interventions including CDT	Varied	Volume (9); Incidence (4)	No	Physiotherapy has a potential effect on secondary lymphedema	PEDro
Rangon [20] critically low	Inception through August 2020	14 (830)	CDT	Multimodal approaches	Volume (14); Pain (2); Symptoms (6); Physical Function (3)	Yes	CDT has a small effect on volume reduction (SMD − 0.18; 95% CI − 0.35, 0.00)	Cochrane risk of bias assessment tool
Smile [21] critically low	January 2006–February 2016	13 (857)	CDT	Unclear	Volume (13)	No	CDT is associated with volume reduction especially in early stage BCRL	None

Abbreviations: *AMSTAR* Assessing Methodology of Systematic Reviews, *BCRL* Breast Cancer-Related Lymphedema, *CDT* Complete Decongestive Therapy, *CI* Confidence Interval, *IPC* intermittent pneumatic compression, *JBI SUMARI* Joanna Briggs Institute System for the Unified Management, Assessment and Review of Information, *MLD* Manual Lymph Drainage, *PNF* Proprioceptive Neuromuscular Facilitation, *QOL* Quality of Life, *SMD* Standard Mean Difference, *UL* Upper Limb

One SR, of critically low quality, indicated that CDT may be most effective in early-stage BCRL [21]. To investigate this finding further, volume outcomes were extracted from the 56 trials included in the SRs where the stage of BCRL could be identified. (Table 3) Of 4 studies that investigated BCRL in patients with mixed staging [22–25], a within group effect size for the intervention could be calculated in 2 studies [22, 24] which ranged from 0.37 to 0.4. In the 4 studies that included participants with mild to moderate BCRL, typically ISL stages I and II, [26–29] the effect size for the intervention group was small (0.3–0.4) in the 2 studies where it could be calculated [27, 29] Moderate stage BCRL was investigated in 2 studies [30, 31] yielding effect sizes of 0.42 and 0.73. Lastly, in the 4 studies investigating moderate-severe BCRL [32–35], effect sizes ranged from 0.6 to 1.6 in the 3 studies where it could be calculated [32, 33, 35]. Overall, in trials where stage of BCRL could be identified, (Table 3) in 12 of these 14 studies the mean volume change for the CDT group exceeded the 150 ml which was identified as the MDC by Taylor et al. [6]. The only 2 trials that did not meet this benchmark were Uzkeser [26] and Luz [28], which both investigated mild-moderate stage lymphedema and reported positive findings short of this metric. This brief investigation of the within group effect size estimation of studies including different stages of lymphedema does not support a greater effect of CDT in those with early-stage disease, but instead indicates a greater volume reduction with later stage disease. These findings are not surprising as the excess volume is higher in patients with more advanced stage disease, but contrasts with the findings of the SR conducted by Smile et al. [21].

**Table 3:** Effect size of complete decongestive therapy by lymphedema stage

Author	Intervention studied	Control	Lymphedema stage	Length/frequency of intervention	Volume change from CDT	CDT effect size (single group)
**Mixed severity**						
McNeely [22]	MLD + Bandaging	Bandaging	Mild-Severe, median duration 39 mo	5x/wk, 4 wks/45 min sessions	**260** (SD 217), 46.1% (SD 22.6)	0.37
Haghighat [23]	CDT + IPC	CDT	3 + mo post-diagnosis; > 50% with stage IIB	5x/wk, 10–15 sessions	**43.1%** mean volume reduction	Unable to calculate
Dayes [24]	Garment + self treatment	CDT (Vodder or Folidi MLD)	Minimum 10% volume difference, 1/3 > 30% Volume difference	5x/wk, 4 wks	**250 ml** (SD 293), EVR 29% (SD 38.6%)	0.4
Hwang [25]	CDT phase 1 and 2	NA	Mixed severity	5xwk/2 wks 10 sessions/daily self Tx and 3 × wk night bandage	1.2% in < 20% EV; **13.1%** EVR in > 20% EV group	Unable to calculate
**Mild-moderate**						
Uzkeser [26]	CDT + IPC	CDT	Stage I-II	5x/wk, 3 wks	150 ml	Unable to calculate
Luz [28]	CDT + Strength Ex	CDT	Stage I-II	2x/wk, 8 wks	119 ml	0.3
Tambour [27]	CDT without MLD	CDT	Mild and Moderate	2x/wk, 3 wks/30–60 min sessions	**151.3** ml, EVR 6.8% (SE 1.2)	0.4
Tastaban [29]	CDT + IPC	CDT	Stage I–II	5x/wk, 4 wks	**170 mL**, EVR 49.6% (48.3–54.9)	Unable to calculate
**Moderate**						
Liao 2013 [30]	CDT	NA	EV 27.7%, Mean duration 22.4 mo ± 16% "Moderate"	Mean 12 sessions (10–26)	**226 ml** (SD 129), EVR 50.5% (SD 23.3)	0.73
Ridner [31]	Laser + Bandaging OR CDT + Laser	CDT with Foldi MLD	12 of 16 stage II	Mean 8 sessions/40 min sessions	EVR **4.9%**	0.42
**Moderate-severe**						
Gradalski [33]	CDT without MLD	CDT (Vodder II)	More advanced (> 20% limb volume difference)	5x/wk, 2 wks	**392 ml**; **47%** relative volume change	1.0
Sen [35]	CDT without MLD	CDT	Stage II and III only	5xwk/3 wks, 45 min	**13.7%** total volume reduction, **56.3%** EVR	1.6
Ergin [34]	CDT + KT	CDT	> 50% severe (> 500 ml excess)	5x/wk, 4 wks	**160.01–228.04** median IQR	unable to calculate
Szuba [32]	CDT (then class II compression garment)	NA	Moderate to Severe	8 ± 3 days or until maximal volume reduction	**298 ml** (SD 257); **44%** (SD 62%) EVR	0.6

Abbreviations: *CDT* Complete Decongestive Therapy, *EVR* Excess Volume Reduction, *IPC* Intermittant Pneumatic Compression, *IQR* Inter Quartile Range, *KT* Kinesiotape, *MLD* Manual Lymphatic Drainage, *Tx* Treatment, *wk* Week

**Bolded** Results met Clinically Important Difference

### Manual lymphatic drainage (MLD)

There were 6 SRs that examined MLD’s effectiveness as an intervention to manage or prevent BCRL (Table 4). Although the primary aim of the SRs reviewed was to determine the effectiveness of MLD on volume reduction in those with BCRL, two examined the effect of MLD on incidence in those at risk of BCRL. Other variables examined in those with BCRL were pain and health-related QOL.

**Table 4:** Systematic reviews of manual lymph drainage as a component of complete decongestive therapy

Author AMSTAR II rating	Time limit of retrieval	Trials number (sample size)	Experimental intervention (number of studies)	Control intervention	Outcomes investigated (number of studies)	Meta-analysis completed	Main conclusion	Quality assessment tool
Lin [17] Moderate	Inception to April 2021	11 (1564)	MLD as a component of CDT (5), alone (1) or with other interventions (Ex, bandaging) (5) Frequency 1–7x/wk 2–52 wks Duration 20 min-1 h	Interventions without MLD	Volume (7) Incidence (3) Pain (3) QOL (4)	Yes	MLD did not significantly impact volume (SMD 0.00; 95% CI – 0.20, 0.21) MLD with exercise reduced incidence of BCRL (RR 0.58; 95% CI 0.37, 0.93) Significant pain reduction (SMD – 0.72; 95%CI – 1.34, – 0.09) No significant change in QOL	Cochrane Risk of Bias Assessment Tool
Müller [11] Moderate	Inception to June 2016	5 (132)	CDT (3) or MLD (2) Frequency 2–5x/wk, 10 sessions-6 wks Duration 5–60 min	CDT without MLD (2), Exercise (1) Laser (1), Electrotherapy (1)	Volume (1) QOL (1)	No	MLD yielded a significant change in volume in only 1 study of CDT MLD improved QOL between groups	Cochrane Risk of Bias Assessment Tool
Qiao [12] Low	Inception to January 2020	8 (236)	MLD alone (2) MLD in CDT (3) or CB (3) Frequency 1–4 wks (8–28 sessions)	Simple lymphatic drainage CDT without MLD or CB alone	Volume (8) Pain (2)	Yes	MLD had no significant increase in volume reduction (SMD 0.43; 95% CI – 0.10, 0.96) No significant reduction in pain (SMD – 0.09; 95% CI – 0.43, 0.25)	Cochrane Risk of Bias Assessment Tool
Thompson [13] Low	Inception to February 2020	17 (869)	CDT with MLD (4) MLD vs other interventions (13) Frequency 5x/wk 2–16 wks (8–48 sessions) Duration 15–60 min	CDT without MLD Exercise, Modalities, Self-MLD	Volume (4) Incidence (4) QOL (11)	No	MLD as a part of CDT did not impact volume outcome MLD had conflicting evidence in reducing incidence No change in QOL with the addition of MLD to other therapies (10)	PEDro
Wanachi [14] Low	January 2000 to June 2020	10 (520)	CDT with MLD (2) MLD with CB (1) MLD with CB (1) Frequency and duration not clearly stated	CDT without MLD CB Standard Treatment with CB	Volume (8) Incidence (2)	No	MLD combined with other treatments yielded no volume improvement in BCRL No additional benefit in reducing incidence of BCRL	Cochrane Risk of Bias Assessment Tool
Liang [16] Critically Low	Inception to May 2019	12 (953)	CDT with MLD (4) CB with MLD (3) MLD with other interventions (modalities, exercise…) (7) Frequency 2–5x/wk 24 days-6 months (10–40 sessions) Duration 15–60 min	Intervention without MLD	Volume (8) Incidence (5)	Yes	Overall: MLD did not improve volume reduction (Pooled SMD – 0.009; 95% CI -0.85, 0.67) *Subgroup Analysis:* Significant volume reduction in patients ≤ 60 years old (SMD: – 1.77; 95%CI – 2.23, – 1.31) and > 1 month intervention (SMD: – 1.77, 95%CI – 2.23, – 1.30] No significant benefit in reducing incidence	AMSTAR guidelines

Abbreviations; *AMSTAR-2* Assessing the Methodological of Systematic Reviews 2, *BCRL* Breast cancer-related lymphedema, *CB* Compression Bandage, *CDT* Complete Decongestive Therapy, *CI* Confidence Interval, *Hr* Hours, *IPC* Intermittent Pneumatic Compression, *MLD* Manual Lymph Drainage, *Min* Minute(s), *PNF* Proprioceptive Neuromuscular Facilitation, *QOL* Quality of Life, *RR* Relative Risk, *SMD* Standardized Mean Difference, *Wk* Week(s), *X* Times

The quality of the SRs examining the effectiveness of MLD ranged from critically low (1 study [16]), low (3 studies [12–14]), and moderate (2 studies [11, 17]). Three SRs [12, 16, 17] completed a meta-analysis on the evidence. The results demonstrate that CDT with the MLD component did not provide further volume reduction as compared to CDT without MLD.

Utilizing MLD as an intervention to prevent BCRL was reviewed by 2 SRs [16, 17] with conflicting results. Lin et al. [17] demonstrated a positive effect of MLD in reducing incidence of BCRL (moderate quality SR) but the SR by Liang et al. [16] (critically low) found no effect of MLD in preventing BCRL. Liang et al. [16] also found that MLD had a positive effect in those younger than 60 years old and when the treatment was greater than 1 month.

Other outcomes used to measure the effect of MLD were pain and health-related QOL. The meta-analysis conducted by Lin 2022 found MLD demonstrated a positive effect on pain reduction. Additionally, a SR by Müller et al. [11], examined the impact of MLD on health-related QOL in those with edema. Within this SR, 5 of the studies focused on breast cancer and were therefore included in this evidence review. The results demonstrated that 1 study [36] showed a positive effect of MLD on health-related QOL in the domains of breast and arm.

Methodologic variability was a challenge throughout all studies included in our review. The diagnosis of BCRL, measurement tool for volume, as well as the interventions varied. As an example, the Lin et al. [4] moderate quality SR, included different stages of BCRL and interventions. The stages of BCRL ranged from at risk, to mild, moderate, and severe. The interventions varied significantly. For example, the intervention studied was CDT with or without MLD [27, 33, 35], MLD with bandaging versus bandaging alone [22, 37], MLD with exercise versus exercise [38], and self or caregiver MLD vs exercise with compression bandage vs observation [39]. The length and frequency also varied from 2 times per week for 3 weeks; 4 times per week for 2 weeks; 5 times a week for either 2, 3, or 4 weeks; and 7 times a week for 4 weeks. Sessions lasted from 20, 30, 45, or 60 min. This variability makes recommending the intervention for each stage, as well as the frequency and duration, challenging at this time. Overall, the MLD component of CDT has no demonstrated benefit in reducing volume. While it may not impact volume, it potentially has impact on other outcomes. Manual lymph drainage may have other significant health effects (pain and QOL) that need to be considered when planning intervention for an individual with BCRL.

### Exercise

Therapeutic exercise is considered an integral aspect of CDT for lymphedema. Three SRs were included with aims focusing on various types of exercise for individuals with or without BCRL when combined with elements of CDT such as MLD (Table 5) [10, 15, 17]. The interventions comprised of aerobic, resistance and aerobic, aquatic therapy, yoga, with the preponderance of evidence in resistive training. The number of training sessions varied between 1 and 7 sessions/week with components of CDT incorporated into the exercise session [10, 15, 17]. The populations within the studies varied in stage of lymphedema and when they initiated the intervention across the continuum of care. Only one SR received a moderate quality rating [10] and one performed a meta-analysis [17]. As exercise has many health benefits, the outcomes investigated included more than arm volume and incidence. These secondary aims included range of motion, pain, strength, arm function, and QOL.

**Table 5:** Exercise as a component of complete decongestive therapy

Author AMSTAR II rating	Time limit of retrieval	Trials number (sample size)	Experimental intervention	Control intervention	Outcomes investigated (number of studies)	Meta-analysis	Main conclusion	Quality assessment tool
Jeffs [10] Moderate	Inception through July 2016	1 (40)	Resistive Ex (1) Frequency 5x/wk for 2 wks then 6wks self-administered resistive ex and CDT	CDT Only	QOL (1)	No	Resistive Ex: Improved physical functioning; body pain; mental health in both groups	JBI SUMARI
Bauman [15] Critically Low	2001–December 2016	11 (458)	All Exercises: Aquatic (2); Yoga (1); DVD home ex with self-massage (1); Supervised Aerobic and Resistive Ex (2); Supervised Resistance Ex (5); Non-supervised Resistive Ex (1); Diaphragmatic breathing; Muscle relaxation; Massage (5) Frequency 10–15 min to 1 h Biweekly to 5x/wk	CDT (2); Non supervised ex (1); Usual care (8)	Volume (11); Pain (2); Muscle Thickness (1); QOL (5); Arm Disability/ Function (2); ROM (2); Weight Loss (1)	No	All Exercises: No significant treatment effect on volume Improved QOL; ROM; Mood; Strength; Muscle Thickness Aquatic Ex: Decreased arm disability Supervised/Non-supervised Resistive Ex. Diaphragmatic breathing, Muscle relaxation, Massage: Improved weight loss	Cochrane Risk of Bias Assessment Tool
Lin [17] Critically Low	No time limit to May 2020	17 (1346)	Shoulder/Elbow Ex (4) Frequency Supervised 1–7x/wk Reps daily to 3–4 x/day Aerobic ex and MLD (4) Frequency 2–5x/wk 30–60 min Aerobic Ex (9) Frequency Supervised 1–2x/wk Unsupervised 3–7x/wk 45–90 min	No supervised ex/Usual care (4) No ex/CDT only (4) No ex/Usual care (9)	Incidence (3); Volume (4); QOL (3); ROM (4); Strength (3); Pain (5); Disability and Symptoms (5)	Yes	Shoulder/Elbow Ex: Reduced incidence of lymphedema (RR 0.343) Aerobic Ex and MLD: No significant difference on volume Aerobic Ex: Improved pain (MD – 1.043); shoulder ROM (flexion MD 3.398; internal rotation MD 3.868); limb dysfunction (MD – 5.231); strength (flexors MD 1.076; abductors MD 0.991)	Jadad Scale

*AMSTAR* Assessing the Methodological Quality of Systematic Reviews, *CDT* Complete Decongestive Therapy, *DVD* Digital Video Disc, *Ex* Exercise, *Hr* Hour, *JBI SUMARI* Joanna Briggs Institute System for the Unified Management, Assessment and Review of Information, *MD* Mean Deviation, *MLD* Manual Lymphatic Drainage, *Min* Minute(s), *RR* Relative Risk; Reps Repetitions, *ROM* Range of Motion, *QOL* Quality of Life

There is limited evidence reporting volume reduction with aerobic or resistive exercise in individuals with BCRL [27, 40–47]. One SR reported early initiation of shoulder and elbow exercise may mitigate risk for developing lymphedema [17]. Additionally, in the studies reviewed, resistance exercise improved QOL, weight loss, range of motion, and strength as well as decreased disability [43–45, 47]. Individuals with BCRL demonstrated improved pain, range of motion and shoulder strength with aerobic exercise as the intervention [41, 42].

Based on the current evidence, exercise should be multimodal to support the musculoskeletal, cardiovascular, and respiratory systems to enhance lymphatic transport and improve symptoms, function, range of motion, and QOL. The individual should receive a pre-exercise assessment and be initially supervised to address comorbidities or impairments as a result of cancer-related treatments. Aerobic and resistive exercises should be tailored to address the individual’s impairments to promote the activation of the muscle pump and enhance lymphatic flow. This includes sequencing proximal to distal, incorporation of diaphragmatic breathing, and slow progression of low-level resistive exercise [43, 45–48]. The evidence supports exercise as safe and efficacious throughout the continuum of care with many health benefits.

### Newer evidence

Between January 2021 and March 2023, 184 additional manuscripts were published on non-surgical or non-pharmacologic approaches to BCRL management. Eleven of these studies directly examined the impact of Phase 1 CDT [49–54], MLD [55–58], and exercise [59] in BCRL (Table 6). Similar to the SR’s, the variability in the methodology in these studies made it challenging to interpret the results.

**Table 6:** Evidence from newer published studies

Author	Study design	Experimental intervention	Control intervention	Stage of disease	Sample size	Outcomes studied	Main conclusion
CDT							
Baran [49]	Pre-post	Phase 1: CDT 5x/week for 3 weeks by a qualified PT 60 min sessions of MLD Foeldi method, CB for 23 h/day, remedial exercises with bandages on, and skin care/education	NA	2	*n* = 27	Volume Skin thickness Sensory testing	↓ volume, ↓ skin thickness, and improvement in sensory testing
Borman [50]	Pre-post	Phase 1: CDT 5x/week for 3 weeks by a CLT MLD × 45 min, exercise × 20 min, skin care/education	NA	1–3	*n* = 50	Volume Function QOL	↓ volume and improvement in function and QOL. Negative relationship between ↓ volume and stage and duration of lymphedema
Bilek [51]	Pre-post	Phase 1: CDT 5x/week for 6 weeks by a CLT MLD, skin care, CB, and remedial exercise	NA	Stage not reported	*n* = 14	Volume Balance Gait assessment	↓ volume and improvement in balance and gait parameters
Corum [52]	Pre-post	Phase 1: CDT to participants with pain CDT 5x/week for 4 weeks by an experienced PT MLD × 30 min, skin care, CB, and resistant exercise	CDT to participants without pain	1–2	n = 45 Pain = 22 No pain = 23	Volume Grip strength Function QOL VAS Pain	↓ volume and improvement in grip strength, function, and QOL in both groups but no difference in groups. Pain decreased in the group with pain
Kostanoglu [53]	Pre-post in older adults	Phase 1: CDT 2x/week for 6 weeks by a CLT MLD × 30 min, CB worn during day only and removed at night, reapplied by a family member who was instructed via video	NA	1–2	*n* = 49	Volume Arm function ADL’s QOL	↓ volume and improvement in arm function, ADL’s, and QOL in older adults with Grade 1 and 2 Adults with Grade 1 had a greater improvement in mobility and participation in life and social activities
Yesil [54]	Pre-post	Phase 1: Participants with edema in the dominant hand received CDT 5x/week for 4 weeks for 1 h/day by a CLT MLD × 30 min. followed by skin care, CB, and remedial exercise	NA	2–3	*n* = 50	Volume Hand kinesthetic Grip strength Function QOL	↓ volume and improvement in QOL, function, grip strength, and kinesthetic sense
MLD							
Devoogdt [55], DeVrieze [56], DeVrieze [57]	RCT	1. CDT using MLD with ICG Phase 1: CDT 5x/week for 3 weeks by an experienced PT All 3 groups received CDT consisting of education, skincare for 5 min, exercise for 10 min, CB for 15 min (worn day and night), and MLD for 30 min. Participant performed self MLD 1x/day, exercise 2x/day, and self skin care and CB on the weekends Phase 2: 18 sessions of additional treatment over 6 months. Daily home program of skin care, custom compression sleeve day and night, ex, self MLD	2. CDT using traditional MLD 3. CDT using MLD placebo consisting of deep massage involving relaxing, transverse movements on the muscles of the ipsilateral neck, back, shoulder, arm, and hand	I, IIa, and IIb	Devoogdt *n* = 187 1. *n* = 62 2. *n* = 62 3. *n* = 63	Lymphatic architecture using lymphofluorscopy	No added value of MLD with ICG based on lymphatic architecture outcomes
					DeVrieze *n* = 182 1. *n* = 60 2. *n* = 61 3. *n* = 61	Volume Excess fluid accumulation in shoulder/trunk Function QOL	↓ volume, ↑ fluid in the shoulder/trunk level, improved function and QOL in all groups but no difference in groups
					DeVrieze *n* = 182 1. *n* = 60 2 *n* = 61 3. *n* = 61	Local tissue water Extracellular fluid Skin thickness Skin elasticity	↓ volume, skin thickness, extracellular fluid, local tissue water, and skin elasticity in the arm in all groups but no difference in groups. Local tissue water, skin thickness, and skin elasticity on the trunk had no difference over time or between groups
Sen [58]	RCT	Phase 1: CDT with MLD for 45–60 min per session 5x/week for 3 weeks (15 sessions)	CDT without MLD	2 and 3	n = 50 (25 in each group)	Volume Function QOL VAS-discomfort, heaviness, and swelling	↓ volume and improvement in function and QOL in both groups but no difference in groups. Addition of MLD decreased discomfort and heaviness symptoms
Exercise							
Zabojszcz [59]	Pre-post	Phase 1: CDT included gymnastics with bandages on for 10 min and 30 min of balance and coordination exercises CDT for 3 weeks. Frequency not reported	NA	Stage not reported	*n* = 30	Balance Postural stability	Improvement in balance and posture

*CB* compression bandaging, *CDT* complete decongestive therapy, *ICG* indocyanine green, *min* minutes, *MLD* manual lymph drainage, *NA* not applicable, *PT* physical therapist, *QOL* quality of life, *VAS* visual analog scale

In the CDT studies, 5 of the 6 studies investigated the impact of CDT 5 times/week using outcome measures beyond volume including grip strength, function, QOL, pain, hand kinesthetic sense, balance, gait, skin thickness, and sensory testing [49–52, 54]. This demonstrates an improvement in other outcomes besides volume which has been portrayed in multiple SRs and trials [11, 36, 43–45, 47]. Borman [50] additionally examined the association of volume reduction with the stage and duration of lymphedema reporting a negative relationship between volume reduction in individuals with more severe and longer duration of lymphedema. This finding is a similar to Smile et al. supporting greater effect of CDT in those with early-stage disease [21]. Kostanoglu et al. [53] examined the use of CDT 2 times/week for 6 weeks in older adults (age range 60–85 years old) with compression bandaging worn during the day only and applied by a family member who was trained via a recording. The study entangled participant characteristics (i.e., older adults) with variation in the treatment approach (frequency, training of person applying compression, duration of wearing compression) making it difficult to extract meaningful results.

Devoogdt et al. [55] and DeVrieze et al. [56, 57] investigated the impact of MLD on CDT by comparing MLD using indocyanine green (ICG) guidance, traditional MLD, and placebo MLD (which consisted of manual treatment on the ipsilateral side). Although this study reported numerous outcomes and thoroughly described the treatment components, all groups received manual treatment making it challenging to interpret the impact of MLD. No significant difference was seen between groups, and thus did not provide support for ICG-guided MLD over more traditional approaches. In a separate study, Sen et al. [58] compared CDT with and without MLD demonstrating the addition of MLD decreased discomfort and heaviness of symptoms, but did not impact excess arm volume. Thus, the more recent evidence likely would not change previous SR’s findings on MLD as a component of CDT.

In the one exercise study, Zabojszcz et al. [59] administered CDT with exercise focused on gymnastics, balance, and coordination exercises demonstrating an improvement in balance and posture. This additional study using balance type exercises in addition to CDT supports the multimodal approach to not only enhance lymphatic transport but also to treat and improve other outcomes such as balance.

The newer evidence demonstrated variability in the methodology which resulted in similar findings as the SR reviews. The variability in outcomes, stage and duration of disease, patient characteristics, frequency and type of treatment, and training of person providing the treatment represented in this new literature continues to make it difficult to determine the full impact of CDT and its components on BCRL.

## Discussion

### Summary

The overall evidence for CDT supports its impact in volume reduction for BCRL. MLD and exercise as separate components of CDT have limited evidence for volume reduction but demonstrate benefits in other outcomes and potentially supports prevention/risk reduction of BCRL. Additional research is needed to study outcome measures beyond volume reduction and identify the essential components of CDT. It will be important to clearly define and consider the population and study characteristics such as stage of disease and phase of treatment in future trials.

The methodology and quality of the SRs makes it challenging to extrapolate the findings to clinical practice. Unfortunately, the results of SRs on CDT are not answering the complexities of the condition because the individual patient’s needs are more complicated than the characteristics that have been studied. Clinically, the individual’s lymphedema etiology, severity, comorbidities, other pertinent clinical information, and individual characteristics are what determines the components of CDT that are applied and the frequency and duration of care. For example, individuals arrive to the clinic at different stages of the disease with different presentations. Only one SR [21] and one newer study [50] dove into a sub-analysis of the efficacy of CDT in relationship to the stage of the disease. Our brief investigation that abstracted volume outcomes from SR trials where stage of BCRL could be identified demonstrated a greater effect on volume in those with later stage disease which contradicts the results of Smile et al. [21]. Given that later stage lymphedema has an increased amount of excess limb volume, our finding is not surprising. Our brief investigation on staging demonstrates the ability to dive deeper into the individual characteristics which would be more clinically applicable to the clinical setting and calls for additional meta-analysis on this topic.

In general, many of the studies focused on investigating ways to shorten the time of treatment and simplify the components of treatment. Considering CDT is the standard of care for this condition, it is critical to start by understanding the impact CDT and each component for the wide variety of populations we serve. Therefore, it is imperative that researchers and clinicians come together on standardizing and reporting on the specific characteristics, such as stages of disease, so future SRs and meta-analysis can generate more specific practice recommendations. In order to allow for comparison across studies, reporting should be standardized even amongst volume outcomes. In our investigation, it was found that some studies report absolute volume changes while others describe changes in excess volume. These differences in reporting could impact the interpretation findings in SRs. Other important individual characteristics that should be considered when constructing research includes demographics, disease duration, and comorbid conditions.

A better understanding of the change in volume that is meaningful for patients is critical. The amount of excess volume reduction required to be impactful could be different based on lymphedema severity, but has not yet been investigated. Differences in patient impact could also be found when looking at variations in volume outcomes such absolute versus excess volume differences, making this another area to be explored. In addition, lymphedema is associated with other symptoms beyond volume such as changes in tissue consistency, reduced function, poorer QOL, and sensation changes. A number of national organizations have made recommendations for BCRL outcome measures [60–64] but the number of outcome measures is quite extensive. Doubblestein and colleagues [65–67] investigated narrowing the choices of BCRL outcome measures and identified 27 that are highly recommended by BCRL experts. This large group of measures cannot, nor should not, be implemented in all cases, so tailoring the measurement approach is still required. By implementing a smaller set of core, standardized BCRL outcomes, selective reporting and variability in use across interdisciplinary groups which manage or research BCRL will be reduced. This will also improve our ability to directly compare the outcomes of different interventions to guide clinical practice.

### Strengths/limitations

This was a comprehensive overview of SRs using well defined criteria for study selection and appraisal on the quality of the SR’s. As a result of identifying the lack of evidence on the stage of disease, we extracted data to investigate the volume outcomes from trials where stage of BCRL could be identified allowing for additional clinically relevant findings. This publication includes only BCRL SRs due to the lack of a large evidence base in populations other than breast cancer. The review identified interventions that impacted upper extremity volume, therefore studies that primarily investigated other outcomes were not included. Articles published in other languages and outside the time frame of this review were not included and are not represented in this publication. Although compression was not included in this review, the compression evidence was presented at the conference by another group.

### Potential future research

The current evidence on effective interventions for BCRL is lacking. Higher quality studies consisting of rigorous, long term, randomized, stratified controlled methods are crucial to help move the field forward. Interventions trials with standard descriptors, including (1) the duration, frequency, intensity, and dose for each component of CDT; (2) individual subject characteristics such as demographics, symptoms, severity of the disease, disease duration, and comorbidities; (3) identification of who is providing the intervention; (4) description about the training and experience on the individual providing the treatment; (5) the treatment regimen; and (6) diagnostic criteria are necessary for stratification analyses and meta-analyses. The current meta-analyses are unsatisfactory and therefore there should be fewer SRs until a higher quality literature pool is available. Future studies using standardized clinically meaningful and feasible outcomes, other than reduction in volume, are critical. Since much of the research focuses on intensive phase CDT in BCRL, investigations of populations other than breast cancer survivors and maintenance phase treatments are needed.

Based on the current literature, there is limited evidence to support CDT for volume reduction in BCRL. With the understanding there is a lack of evidence due to the inadequacy of optimally conducted clinical trials, more work is needed focusing on untangling the efficacy of treatment through high quality studies. A consensus document that integrates this evidence with expert and patient viewpoints will assist in guiding practice to provide optimal care.

## Supplementary Information

Below is the link to the electronic supplementary material.

Supplementary file 1 (PDF 88 kb)

## Data Availability

Data from this review are available in the supplemental table and by reasonable request to the corresponding author.
