# Supplementary material for: Effectiveness of complete decongestive therapy for upper extremity breast cancer-related lymphedema: a review of systematic reviews
Source: Med Oncol. 2024 Oct 23;41(11):297. doi: 10.1007/s12032-024-02421-6 (PMC11496316; doi:10.1007/s12032-024-02421-6)
Supplement: Supplementary file 1 — (PDF 88 kb) [file 12032_2024_2421_MOESM1_ESM.pdf]

Individual Study Author Year	Experimental Intervention	Control Intervention	Prevention / Intervention	Length/ Frequency of Intervention	**Qiao 2023;** Meta-Analysis MLD for Volume and # Treatments	**Lin 2023;** Meta-analysis Exercise on volume and QOL	**Rangon 2022;** Meta-Analysis CDT for Volume and Symptoms	**Lin 2022;** Meta anlysis for effect of MLD on symptom, volume, QOL	**Wanchai 2021;** SR MLD for Volume	**Naik 2021;** SR CDT Any outcome	**Thompson 2020;** SR MLD for Volume, Symptoms, Quality of Life	**Lytvyn 2020;** Meta-Analysis CDT on Volume	**Liang 2020;** Meta-Analysis MLD for Volume and prevention of BCRL	**Smile 2018;** CDT SR on V, S, QOL	**Muller 2018;** SR MLD on QOL Outcomes	**Jeffs 2018;** SR of CDT and Exercise on volume and patient centered outcomes	**Baumann 2018;** SR of exercise on volume and symptoms; CDT articles*
Johansson 1998	MLD with compression sleeve	Sequential pneumatic compression with compression sleeve	Intervention, median duration 14 mo	5x/wk, 2 wks/ 45 min session	V						V, S		V				
Johanssen 1999	MLD+ CB	CB	Intervention	MLD+CB 5 days / CB 3wks	V								V				
Andersen 2000	MLD + Bandaging	Bandaging	Intervention, median duration 12 mo	4x/wk, 2 wks /60 min sessions	V			V	V		V, S	V	V	V			
Sitzia 2002	MLD with compression bandaging	simplified MLD with compression bandaging	Intervention	5x/ wk, 2 weeks/ 40 - 80 mins sessions	V				V		V	V	V				
Szuba 2002	CDT+IPC	CDT	Intervention, mean duration 41.1 mo	IPC pressure 40-50 mmHg, 30 min/session, 10 d								V					
Williams 2002	MLD and compression sleeves	Self MLD with compression sleeve	intervention	5x/wk, 3 wks / 45 min sessions	V	V			V		V, S		V				
McNeely 2004	MLD + Bandaging	Bandaging	Intervention, median duration 39 mo	5x/wk, 4 weeks / 45 min sessions	V			V	V		V	V	V			V	
Johansson 2005	Compression w/ low intensity exercise	Low intensity Exercise	Intervention	not stated						V, S, O (Physical Activity)							
Didem 2005	CDT with MLD (Foldi)	CDT without MLD	Intervention, median duration 31.6 mo	3x/wk, 4 weeks			V					O (DASH, SH ABD)	V				
McNeely 2009	MLD + compression	Compression	Intervention	4 weeks						V							
Szolnoky 2009	CDT + IPC	CDT	Intervention, mean duration 16.3 mo	5x/wk, 2 weeks; Pressure 40-50 mm Hg, 30 min sessions			V, S		V			S	V				
Wigg 2009	CDT with either MLD vs CP		Intervention, 1+ yr, most moderate									V					
Lacomba 2010	MLD, massage of scar tissue; AROM of ex; education	Education only	Intervention	not stated						V							
Haghighat 2010	CDT + IPC	CDT	Intervention, 3+ mo >50% with stage IIB	5x/wk, 10-15 sessions			S					S					
Kim 2010	CDT + Resistance Ex	CDT	Intervention, 5.3 mo post-dx, no staging	5x/wk, 2 weeks			V									QOL	V, QOL
Sarri 2010	Physiotherapeutic stimulation using Foldi technique	No Physiotherapeutic stimulation	Intervention	not stated						O (Lymphatic Flow)							
Bongi 2011	MLD	Observation	Intervention	60 min, 1X week, 5 weeks											QOL		
Devoogdt 2011	MLD+ Ex	Exercise	Prevention	1-3x wk to 1x wk for 40 sessions/ 30 mins per session				V, QOL	V	V	V		V				

Belmonet 2011	MLD	Low frequency low intensity electrotherap y	Intervention	5x/wk, 2 wks/ no time of sessions							V, S, QOL		V		QOL		
Uzkeser 2011	CDT + CP	CDT	Intervention, Stage I or II									V					
Gurdal 2012	CDT	IPC + SLD	Intervention, mild-severe Stillwell classification	3x weekly, 6 weeks					V			QOL		V			
Maher 2012	MLD	MLD + Compression Bandaging	Intervention						V								
Zimmermann 2012	MLD + exs +chest PT	ex +chest PT+self drainage	intervention	5xwk/ 2wks, then 2x wk from day 14 to 6 mos					V	V	V		V				
Dayes 2013	CDT (Vodder or Folidi MLD)	Garment + Self treatment	Intervention, minimum 10% volume difference, 1/3 >30% Volume difference	5x/wk, 4 weeks	V		V, O (Physical Function)					V, QOL		V, QOL, O (arm function)	QOL	V, QOL, O (limb function)	
Haghighat 2013	CDT (Vodder MLD) phase 1		Intervention	5x wk/ 2-3 weeks 12 to 15 sessions										V, QOL, O (arm function)		V, S	
Hwang 2013a	CDT phase 1 and 2	NA	Intervention	5xwk/ 2 wks 10 sessions/ daily self Rx and 3 x wk nightbandage										V		V	
Ridner 2013	MLD Foldi + Bandaging	Laser + Bandaging OR MLD + Laser + Bandaging	Intervention, 12 of 16 stage II	Ave 8 sessions / 40 min sessions							V, S		V	V, O (skin)	QOL		
Vignes 2013	CDT 11 days vs. 4 days		intervention, median onset 12 months	11 day vs 4 day										V			
Liao 2013	Efficacy of CDT on BCRL		intervention, mean duration 22.4 mo, PEV 27.7% +/-16% "moderate"	mean 12 sessions (10-26)										V			
Bergmann 2014	CDT +/- MLD		Intervention	3x/week until plateau 9-12 sessions / 30 mins session	V		S			V, S, O (Shoulder Function)	V, O (theraputic response)	V, S	V	V			
Odebiyi 2014	MLD+exs v exe		intervention	2x/wk, 6 wks/ 15 min sessions							V, QOL, O (fatigue)				QOL		
Pekyavas 2014	CDT +/- KT		Intervention	5x/wk, 2 weeks			S							?			
Buragadda 2015	CDT: garment type + Ex		Intervention, unclear stage	5x/wk, 6 weeks			V, S, O (Physical Function)					V		V, S, O (arm function)			
Do 2015	CDT +/- Resistance Ex	CDT + Resistance Ex	Intervention	5x/wk, 1 or 2 weeks			V, O (Physical Function)					P, QOL, O (DASH, SH ABD)					
Gradalski 2015	CDT +/- MLD (Vodder II)		Intervention, more advanced (>20% limb volume difference)	5x/wk, 2 weeks			V, S	V, QOL	V		V, QOL	V, S, QOL, O (Fatigue)		V	QOL	V, QOL, O (perception)	
Uzkeser 2015	CDT +/- IPC		Intervention, stage 1-2	5x/wk, 3 weeks			S							V			
Atalay 2015	CDT		Intervention	unspecified										V, O (AROM, depression)			
Kim 2015	CDT vs stellate ganglion block		intervention	follow up at 2, 4 and 8 week										V			V, QOL
Cho 2016	MLD + Ex vs Ex		Intervention AWS	5x/wk, 4wks / 30 mins sessions				V, S, QOL, O (shoulder function)		V, S, QOL, O (Shoulder Function)	S, O (incidence)		V	V,S			

Bok 2016	CDT + Resistance	CDT	Intervention									V					V, O (subcutaneous thickness, Muscle thickness)
Zhang 2016	MLD + Ex	Ex	Prevention	3x/day/from 10 - 30 days after surgery				V, O (shoulder fxn)					V				
Liao 2016	Efficacy of CDT on malignant lymphedema													V			
Domez 2017	Physical activity and simple lymphatic drainage	No intervention	Intervention	not stated						S, O (Physical Activity, UE Problems)							
Do 2017	CDT + CPS	DT + CPS + RI	Intervention									S, QOL, O (Fatigue)					
Ha 2017	MLD + PNF vs PNF		Intervention	3x wk, 16 wks, 30 mins sessions				No MA: V, S, O(shoulder function, depression)			V, S						
Park 2017	Active and Resistive Exercise	CDT	Intervention									S, O (SH ABD)					
Devoogdt 2018	MLD + Exercise	Exercise	Prevention	1-3x/wk, decrease to 1x/wk for 6 mos				V, QOL, O (shoulder fxn)		V, QOL, O (ROM)	O (incidence, functioning)		V				
Tambour 2018	CDT	CDT without MLD	Intervention, mild & moderate	2x/wk, 3 weeks / 30 - 60 min sessions	V	V	V, S	V, S, QOL			V, O (shoulder tension)	V, S, O (ADL and Return to Work)	V				
Luz 2018	CDT + Resistance	CDT										O (DASH, SH ABD)					
Ergin 2019	CDT +/- KT		Intervention, >50% severe (>500 ml excess)	5x/wk, 4 weeks			V										
Sanal-Toprak 2019	MLD+CB V IPC (30 mins) +CB		intervention, stage 2 & 3	3x/wk, 5wks, 30 mins sessions							V, S, O (shoulder ROM)	0 (SH ABD)					
Ligabue 2019	Self-MLD	Usual Care (education)	maintenance									V					
Pasyar 2019	Yoga + Routine Care including CDT and self-MLD	Routine Care including CDT and Self MLD				V						V, S, QOL, O (Fatigue)					
Gol 2020	MLD	Observation	Prevention	7x/wk, 4 wks, 20 mins sessions				V, S									
Tastaban 2020	CDT + IPC	CDT	Intervention, Stage I and II	5x/wk, 4 weeks			S, O (Physical Function)										
Sen 2020 (2021)	CDT +/- MLD		Intervention, ISL stage 2 and 3 only	5xwk/3 wks, 45 mins				V, QOL									
